# Crystal structure of poly[μ_2_-aqua-aqua­(μ_2_-4-nitro-2,5,6-trioxo-1,2,5,6-tetra­hydro­pyridin-3-olato)hemi-μ_4_-oxalato-barium(II)]

**DOI:** 10.1107/S2056989015006520

**Published:** 2015-04-09

**Authors:** Rusul Alabada, Olga Kovalchukova, Irina Polyakova, Svetlana Strashnova, Vladimir Sergienko

**Affiliations:** a Al Muthanna University, Samawah, Al Mutanna, Iraq; b Peoples’ Friendship University of Russia, 6 Miklukho-Mallaya, 117198 Moscow, Russian Federation; c Kurnakov Institute of General and Inorganic Chemistry RAS, 31 Leninskiy Avenue, 119991 Moscow, Russian Federation

**Keywords:** crystal structure, two-dimensional coordination polymer, barium(II) compound, oxalate

## Abstract

In the title coordination polymer, [Ba(C_5_HN_2_O_6_)(C_2_O_4_)_0.5_(H_2_O)_2_]_*n*_, the tenfold coordination of the Ba centre consists of four O atoms from the two 4-nitro-2,5,6-trioxo-1,2,5,6-tetra­hydro­pyridin-3-olate (*L*) anions, three O atoms of two oxalate anions and three water mol­ecules. The Ba—O bond lengths fall in the range 2.698 (3)–2.978 (3) Å. The *L* ligand chelates two Ba atoms related by a screw axis, leading to formation of fused five- and six-membered chelate rings. Due to the bridging function of the ligands and water mol­ecules, the complex monomers are connected into polymeric two-dimensional layers parallel to the *bc* plane. Inter­molecular O—H⋯O hydrogen bonds link these layers into a three-dimensional supra­molecular framework.

## Abstract 2

In a Ba coordination polymer incorporating 4-nitro-2,5,6-trioxo-1,2,5,6-tetra­hydro­pyridin-3-olate, oxalate and aqua ligands, the complex monomers are connected into polymeric two-dimensional layers parallel to the *bc* plane. Inter­molecular O—H⋯O hydrogen bonds link these layers into a three-dimensional supra­molecular framework.

## Chemical context   

Mixed ligand coordination polymers containing bridging oxalate anions and 1,2-dicarbonyl carbocyclic or heterocyclic compounds exhibit high reactivity and different types of magnetism (Aldoshin, 2008 ▸; Coronado *et al.*, 2007 ▸; Kitagawa & Kawata, 2002 ▸; Kovalchukova & Strashnova, 2014 ▸; Ohba & Okawa, 2000 ▸). Such compounds are of chemical inter­est, since a large number of potential donors available in the ligands predetermine a variety of coordination modes, which afford different geometries and dimensionalities of coordination polymers. Recently, we reported the synthesis, crystal structure and some properties of metal complexes of the 2,3,5,6-tetra­oxo-4-nitro-4-idene anion (Kovalchukova *et al.*, 2014 ▸, 2014 ▸; Dinh Do *et al.*, 2013 ▸). The above-mentioned anion does not replace the water mol­ecules from the inner sphere of the hydrated metal cations [*M*(H_2_O)_6_]^*n*+^, but can coordinate metal centres like sodium and silver(I). In the present paper, we report the mol­ecular and crystal structure of a mixed-ligand barium complex containing 4-nitro-2,5,6-trioxo-1,2,5,6-tetra­hydro­pyridin-3-olate (*L*) and oxalate anions as ligands.
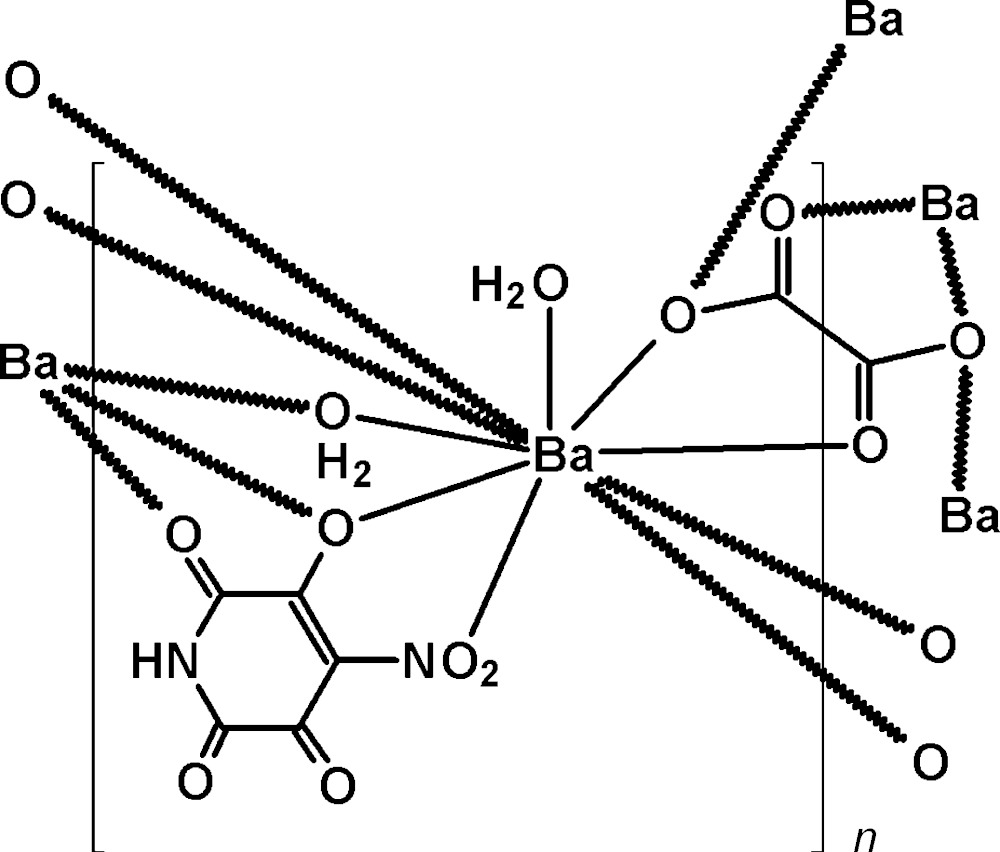


## Structural commentary   

**Figure 1:**
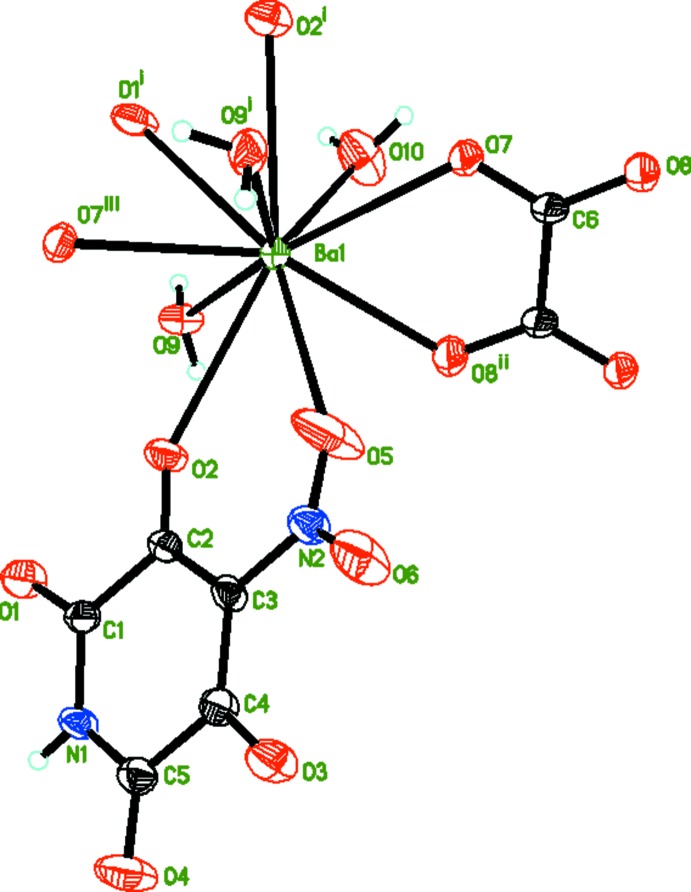
View of (I), showing the atom-labelling scheme and 50% probability displacement ellipsoids. [Symmetry codes: (i) −*x*, *y* + 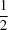, −*z* + 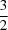; (ii) −*x*, −*y* + 1, −*z* + 1; (iii) −*x*, *y* − 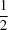, −*z* + 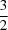.]

In the title compound, [Ba(C_5_HN_2_O_6_)(C_2_O_4_)_0.5_(H_2_O)_2_]_*n*_, (I) (Fig. 1 ▸), the tenfold coordination of the Ba1 atom (Table 1 ▸) is formed by the O2, O5, O1 and O2 atoms of two 4-nitro-2,3,5,6-tetra­oxo­pyridine-4-ide anions (*L*), the O7, O8 and O7A atoms of two oxalate anions, and the O9, O9A and O10 atoms of water mol­ecules. The Ba—O bond lengths fall in the range 2.698 (3)–2.978 (3) Å, which is typical for ten-coordinate barium complexes containing oxalate anions (Viciano-Chumillas *et al.*, 2010 ▸; Heinl *et al.*, 2002 ▸; Marinescu *et al.*, 2005 ▸; Belombe *et al.*, 2003 ▸, 2012 ▸; Larsson, 2001 ▸; Bouayad *et al.*, 1995 ▸; Iveson *et al.*, 2011 ▸). The *L* anion has a flattened skeleton. The r.m.s. deviation of the six ring atoms from their mean plane is 0.0256 Å; the O2 and O4 atoms lie in this plane deviating by 0.049 (3) and 0.010 (3) Å, respectively, whereas the O1 and O3 atoms deviate from it by 0.171 (3) and 0.077 (3) Å, respectively. The plane of the nitro group is rotated by 11.9 (8)° with respect to the ring plane. The *L* ligand chelates two Ba atoms related by a screw axis forming fused chelate rings. The six-membered ring is almost planar (r.m.s. deviation = 0.0353 Å) and the five–membered ring is folded along the O1—O2 line by 19.0°. The geometry of the *L* anion in the Ba complex is close to that in the compounds studied earlier (Kovalchukova *et al.*, 2014 ▸; Dinh Do *et al.*, 2013 ▸). All C=O bonds are of the double-bond type [1.200 (5)–1.229 (5) Å]. The monodentate coordination of *L via* the O atom of a nitro group attached to a benzene ring is in accordance with Venkatasubramanian *et al.* (1984 ▸), Harrowfield *et al.* (1998 ▸) and Chantrapromma *et al.* (2002 ▸). The centrosymmetric oxalate anion connects four Ba atoms closing two almost planar five–membered rings (r.m.s. deviation of rings = 0.0415 Å). 

## Supra­molecular features   

**Figure 2:**
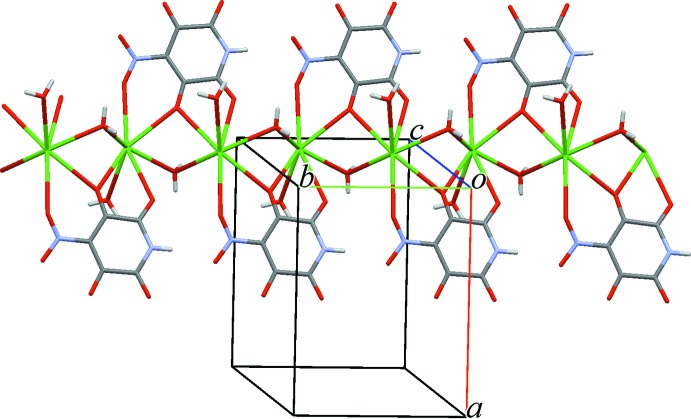
One-dimensional polymeric chain in (I). Oxalato ligands omitted for clarity.

**Figure 3:**
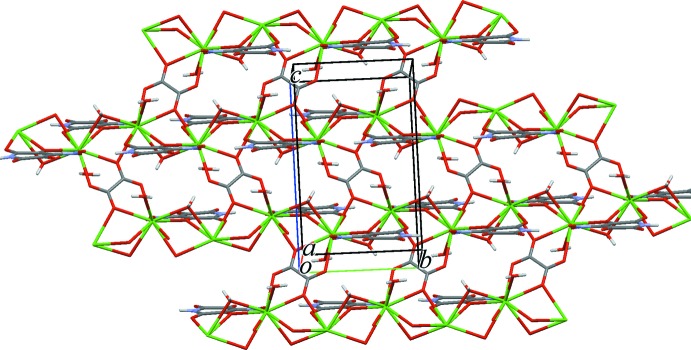
two-dimensional polymeric layer in (I) viewed approximately along the *a* axis.

Due to the bridging function of the *L* ligand and the O9 water mol­ecule, the hydrated complex cations [Ba(*L*)(H_2_O)_2_]^+^ form wide zigzag bands running along the screw axes in the *b*-axis direction (Fig. 2 ▸). Oxalate anions connect the bands into thick two-dimensional networks parallel to *bc*. The networks have corrugated surfaces with terminal O3, O4 and O6 atoms of the *L* ligand on the ‘hills’ and water mol­ecules in the ‘hollows’. In the packing (Fig. 3 ▸), the ‘hills’ enter the ‘hollows’ of adjacent networks. Two-centre hydrogen bonds O9—H2⋯O3 and O10—H5⋯O4 and three-centre bonds O10—H4⋯(O3,O6) (Table 2 ▸) link the networks into a three-dimensional framework. Hydrogen bonds N1—H1⋯O6 and O9—H3⋯O8 link the elements of a band and a network, respectively.

## Database survey   

The synthesis, crystal structure and some properties of metal complexes of the 4-nitro-2,3,5,6-tetraoxo-4-idene anion are described in Kovalchukova *et al.* (2014 ▸) and Dinh Do *et al.* (2013 ▸). Model structures of complexes containing carbocyclic polyoxo compounds are reviewed in Kitagawa & Kawata (2002 ▸) and Kovalchukova & Strashnova (2014 ▸). Ten coordinated structures of Ba cations with oxalate anions containing other *O*-donating ligands have been described (Viciano-Chumillas *et al.*, 2010 ▸; Marinescu *et al.*, 2005 ▸; Belombe *et al.*, 2003 ▸, 2012 ▸; Larsson, 2001 ▸; Bouayad *et al.*, 1995 ▸; Iveson *et al.*, 2011 ▸). Monodentate coordination *via* the O atom of a nitro aromatic group is described by Venkatasubramanian *et al.* (1984 ▸), Harrowfield *et al.* (1998 ▸) and Chantrapromma *et al.* (2002 ▸).

## Synthesis and crystallization   

Single crystals of (I) were grown by the slow evaporation of an ethanol solution of a 1:1:1 molar mixture of barium chloride, ammonium oxalate and ammonium 2,3,5,6-tetraoxo-4-nitro-4-inide.

## Refinement details   

Crystal data, data collection and structure refinement details are summarized in Table 3 ▸. The H atoms of water mol­ecules were localized in a difference map; O—H distances were normalized. The position of the amino H atom was calculated. All H atoms were refined within the riding model, with *U*_iso_(H) = 1.2*U*_eq_ of the parent atom. The crystal studied was a twin without superposition of reciprocal lattices. Apparently, an accidental overlapping of reflections of two domains is responsible for increased displacement ellipsoids of some atoms. The *U*_*ij*_ components of atom O5 were restrained to approximate the isotropic behaviour.

## Supplementary Material

Crystal structure: contains datablock(s) I, global. DOI: 10.1107/S2056989015006520/cv5485sup1.cif

Structure factors: contains datablock(s) I. DOI: 10.1107/S2056989015006520/cv5485Isup2.hkl

Supporting information file. DOI: 10.1107/S2056989015006520/cv5485Isup3.mol

CCDC reference: 1057170

Additional supporting information:  crystallographic information; 3D view; checkCIF report

## Figures and Tables

**Table 1:** Selected bond lengths ()

Ba1O8^i^	2.698(3)	Ba1O10	2.882(4)
Ba1O7	2.728(3)	Ba1O1^iii^	2.889(3)
Ba1O9	2.755(3)	Ba1O5	2.914(4)
Ba1O7^ii^	2.805(3)	Ba1O2	2.931(3)
Ba1O9^iii^	2.860(3)	Ba1O2^iii^	2.978(3)

Symmetry codes: (i) 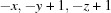; (ii) 
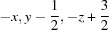
; (iii) 
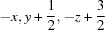
.

**Table 2:** Hydrogen-bond geometry (, )

*D*H*A*	*D*H	H*A*	*D* *A*	*D*H*A*
N1H1O6^i^	0.86	2.21	3.052(5)	166
O9H2O3^ii^	0.82	2.10	2.877(4)	158
O9H3O8^i^	0.82	1.84	2.639(4)	163
O10H4O3^ii^	0.82	2.14	2.828(5)	141
O10H4O6^ii^	0.82	2.30	3.010(5)	146
O10H5O4^iii^	0.82	2.41	3.210(5)	165

Symmetry codes: (i) 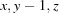; (ii) 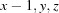; (iii) 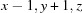.

**Table 3:** Experimental details

Crystal data	
Chemical formula	[Ba(C_5_HN_2_O_6_)(C_2_O_4_)_0.5_(H_2_O)_2_]
*M*_r_	804.92
Crystal system, space group	Monoclinic, *P*2_1_/*c*
Temperature (K)	296
*a*, *b*, *c* ()	10.3283(11), 7.9868(8), 13.0760(14)
()	96.419(2)
*V* (^3^)	1071.88(19)
*Z*	2
Radiation type	Mo *K*
(mm^1^)	3.76
Crystal size (mm)	0.16 0.12 0.03

Data collection	
Diffractometer	Bruker APEXII CCD
Absorption correction	Multi-scan (*SADABS*; Bruker, 2004 ▸)
*T*_min_, *T*_max_	0.586, 0.746
No. of measured, independent and observed [*I* > 2(*I*)] reflections	11240, 3410, 2692
*R*_int_	0.036
(sin /)_max_ (^1^)	0.736

Refinement	
*R*[*F* ^2^ > 2(*F* ^2^)], *wR*(*F* ^2^), *S*	0.038, 0.093, 1.05
No. of reflections	3410
No. of parameters	172
No. of restraints	6
H-atom treatment	H-atom parameters constrained
_max_, _min_ (e ^3^)	2.31, 1.67

Computer programs: *APEX2* and *SAINT* (Bruker, 2004 ▸), *SHELXS97* and *SHELXTL* (Sheldrick, 2008 ▸), *SHELXL2013* (Sheldrick, 2015 ▸), *ORTEP* (Johnson Burnett, 1996 ▸) and *Mercury* (Macrae *et al.*, 2008 ▸).

## supplementary crystallographic information

### Crystal data

**Table:**

[Ba(C_5_HN_2_O_6_)(C_2_O_4_)_0.5_(H_2_O)_2_]	*F*(000) = 764
*M**_r_* = 804.92	*D*_x_ = 2.494 Mg m^−^^3^
Monoclinic, *P*2_1_/*c*	Mo *K*α radiation, λ = 0.71073 Å
*a* = 10.3283 (11) Å	Cell parameters from 3977 reflections
*b* = 7.9868 (8) Å	θ = 3.0–31.4°
*c* = 13.0760 (14) Å	µ = 3.76 mm^−^^1^
β = 96.419 (2)°	*T* = 296 K
*V* = 1071.88 (19) Å^3^	Plate, brown
*Z* = 2	0.16 × 0.12 × 0.03 mm

### Data collection

**Table:**

Bruker APEXII CCD diffractometer	2692 reflections with *I* > 2σ(*I*)
Radiation source: sealed tube	*R*_int_ = 0.036
φ and ω scans	θ_max_ = 31.5°, θ_min_ = 3.0°
Absorption correction: multi-scan (*SADABS*; Bruker, 2004)	*h* = −15→14
*T*_min_ = 0.586, *T*_max_ = 0.746	*k* = −11→11
11240 measured reflections	*l* = −19→18
3410 independent reflections	

### Refinement

**Table:**

Refinement on *F*^2^	Primary atom site location: structure-invariant direct methods
Least-squares matrix: full	Secondary atom site location: difference Fourier map
*R*[*F*^2^ > 2σ(*F*^2^)] = 0.038	Hydrogen site location: mixed
*wR*(*F*^2^) = 0.093	H-atom parameters constrained
*S* = 1.05	*w* = 1/[σ^2^(*F*_o_^2^) + (0.0492*P*)^2^ + 0.9998*P*] where *P* = (*F*_o_^2^ + 2*F*_c_^2^)/3
3410 reflections	(Δ/σ)_max_ < 0.001
172 parameters	Δρ_max_ = 2.31 e Å^−^^3^
6 restraints	Δρ_min_ = −1.67 e Å^−^^3^

### Special details

**Table:**

Geometry. All e.s.d.'s (except the e.s.d. in the dihedral angle between two l.s. planes) are estimated using the full covariance matrix. The cell e.s.d.'s are taken into account individually in the estimation of e.s.d.'s in distances, angles and torsion angles; correlations between e.s.d.'s in cell parameters are only used when they are defined by crystal symmetry. An approximate (isotropic) treatment of cell e.s.d.'s is used for estimating e.s.d.'s involving l.s. planes.

### Fractional atomic coordinates and isotropic or equivalent isotropic displacement parameters (Å^2^)

**Table:**

	*x*	*y*	*z*	*U*_iso_*/*U*_eq_	
Ba1	−0.00361 (2)	0.20711 (3)	0.69482 (2)	0.01991 (8)	
O1	0.2105 (3)	−0.4097 (4)	0.6917 (3)	0.0343 (8)	
O2	0.1678 (3)	−0.0830 (4)	0.6825 (3)	0.0295 (7)	
O3	0.6026 (3)	−0.0297 (4)	0.6022 (3)	0.0344 (8)	
O4	0.6113 (4)	−0.3626 (5)	0.5935 (4)	0.0489 (10)	
O5	0.2660 (4)	0.2124 (5)	0.6490 (5)	0.0711 (16)	
O6	0.4673 (4)	0.2379 (4)	0.6424 (4)	0.0446 (10)	
O7	−0.0517 (3)	0.5240 (4)	0.6225 (2)	0.0259 (6)	
O8	−0.0324 (4)	0.7141 (4)	0.5006 (3)	0.0380 (9)	
O9	−0.1222 (3)	−0.0942 (4)	0.6412 (2)	0.0277 (7)	
H2	−0.2018	−0.1018	0.6364	0.033*	
H3	−0.1064	−0.1457	0.5898	0.033*	
O10	−0.2582 (4)	0.2730 (5)	0.5886 (4)	0.0452 (10)	
H4	−0.3231	0.2206	0.6007	0.054*	
H5	−0.2776	0.3726	0.5862	0.054*	
N1	0.4071 (3)	−0.3879 (4)	0.6348 (3)	0.0236 (7)	
H1	0.4119	−0.4950	0.6295	0.028*	
N2	0.3712 (4)	0.1478 (5)	0.6435 (3)	0.0258 (7)	
C1	0.2940 (4)	−0.3217 (5)	0.6640 (3)	0.0211 (8)	
C2	0.2754 (4)	−0.1295 (5)	0.6620 (3)	0.0196 (7)	
C3	0.3832 (4)	−0.0309 (5)	0.6406 (3)	0.0201 (7)	
C4	0.5038 (4)	−0.1026 (5)	0.6192 (3)	0.0217 (8)	
C5	0.5130 (4)	−0.2959 (6)	0.6135 (4)	0.0250 (8)	
C6	−0.0251 (4)	0.5680 (5)	0.5350 (3)	0.0218 (8)	

### Atomic displacement parameters (Å^2^)

**Table:**

	*U*^11^	*U*^22^	*U*^33^	*U*^12^	*U*^13^	*U*^23^
Ba1	0.02335 (13)	0.01750 (12)	0.01979 (12)	0.00255 (10)	0.00648 (8)	0.00287 (9)
O1	0.0313 (17)	0.0196 (16)	0.056 (2)	−0.0044 (13)	0.0208 (15)	0.0017 (14)
O2	0.0223 (15)	0.0213 (16)	0.048 (2)	0.0046 (12)	0.0156 (13)	0.0036 (13)
O3	0.0191 (15)	0.0244 (17)	0.061 (2)	−0.0021 (13)	0.0110 (14)	0.0034 (15)
O4	0.0296 (18)	0.0305 (19)	0.091 (3)	0.0069 (16)	0.0260 (19)	0.000 (2)
O5	0.037 (2)	0.026 (2)	0.157 (5)	0.0086 (17)	0.038 (3)	0.007 (2)
O6	0.0302 (18)	0.0179 (17)	0.087 (3)	−0.0070 (14)	0.0102 (19)	0.0005 (18)
O7	0.0408 (18)	0.0192 (14)	0.0193 (14)	0.0009 (13)	0.0101 (13)	−0.0011 (11)
O8	0.068 (3)	0.0215 (16)	0.0281 (17)	0.0077 (17)	0.0221 (17)	0.0028 (13)
O9	0.0241 (15)	0.0280 (16)	0.0318 (16)	0.0028 (13)	0.0063 (12)	−0.0083 (13)
O10	0.0294 (18)	0.032 (2)	0.072 (3)	−0.0050 (16)	−0.0021 (18)	0.0092 (18)
N1	0.0228 (17)	0.0139 (15)	0.035 (2)	0.0023 (13)	0.0096 (14)	0.0000 (14)
N2	0.0228 (17)	0.0192 (17)	0.036 (2)	0.0005 (14)	0.0066 (15)	0.0007 (15)
C1	0.0216 (19)	0.0184 (19)	0.0245 (19)	0.0014 (14)	0.0075 (15)	0.0000 (14)
C2	0.0198 (18)	0.0168 (18)	0.0227 (18)	0.0023 (15)	0.0049 (14)	0.0005 (15)
C3	0.0187 (18)	0.0146 (17)	0.027 (2)	−0.0003 (14)	0.0032 (15)	0.0012 (14)
C4	0.0191 (18)	0.0180 (18)	0.028 (2)	−0.0019 (15)	0.0043 (15)	0.0003 (15)
C5	0.0211 (19)	0.024 (2)	0.031 (2)	0.0023 (17)	0.0056 (16)	0.0027 (17)
C6	0.027 (2)	0.0183 (19)	0.0211 (19)	0.0020 (15)	0.0073 (15)	−0.0044 (14)

### Geometric parameters (Å, º)

**Table:**

Ba1—O8^i^	2.698 (3)	O7—C6	1.257 (5)
Ba1—O7	2.728 (3)	O7—Ba1^iii^	2.805 (3)
Ba1—O9	2.755 (3)	O8—C6	1.250 (5)
Ba1—O7^ii^	2.805 (3)	O8—Ba1^i^	2.698 (3)
Ba1—O9^iii^	2.860 (3)	O9—Ba1^ii^	2.860 (3)
Ba1—O10	2.882 (4)	O9—H2	0.8200
Ba1—O1^iii^	2.889 (3)	O9—H3	0.8200
Ba1—O5	2.914 (4)	O10—H4	0.8201
Ba1—O2	2.931 (3)	O10—H5	0.8200
Ba1—O2^iii^	2.978 (3)	N1—C5	1.372 (5)
O1—C1	1.199 (5)	N1—C1	1.375 (5)
O1—Ba1^ii^	2.889 (3)	N1—H1	0.8600
O2—C2	1.229 (5)	N2—C3	1.433 (5)
O2—Ba1^ii^	2.978 (3)	C1—C2	1.547 (6)
O3—C4	1.217 (5)	C2—C3	1.417 (5)
O4—C5	1.202 (5)	C3—C4	1.427 (5)
O5—N2	1.212 (5)	C4—C5	1.549 (6)
O6—N2	1.227 (5)	C6—C6^i^	1.546 (8)
			
O8^i^—Ba1—O7	59.61 (9)	O2—Ba1—O2^iii^	149.14 (6)
O8^i^—Ba1—O9	93.86 (10)	C1—O1—Ba1^ii^	124.3 (3)
O7—Ba1—O9	131.58 (10)	C2—O2—Ba1	144.8 (3)
O8^i^—Ba1—O7^ii^	153.12 (10)	C2—O2—Ba1^ii^	122.0 (3)
O7—Ba1—O7^ii^	142.27 (8)	Ba1—O2—Ba1^ii^	91.81 (8)
O9—Ba1—O7^ii^	78.67 (9)	N2—O5—Ba1	152.8 (3)
O8^i^—Ba1—O9^iii^	118.77 (10)	C6—O7—Ba1	121.7 (3)
O7—Ba1—O9^iii^	78.16 (9)	C6—O7—Ba1^iii^	125.8 (3)
O9—Ba1—O9^iii^	145.81 (7)	Ba1—O7—Ba1^iii^	100.18 (9)
O7^ii^—Ba1—O9^iii^	67.59 (9)	C6—O8—Ba1^i^	123.3 (3)
O8^i^—Ba1—O10	73.50 (13)	Ba1—O9—Ba1^ii^	98.17 (9)
O7—Ba1—O10	62.78 (10)	Ba1—O9—H2	119.8
O9—Ba1—O10	71.41 (10)	Ba1^ii^—O9—H2	112.6
O7^ii^—Ba1—O10	126.42 (11)	Ba1—O9—H3	121.6
O9^iii^—Ba1—O10	125.00 (11)	Ba1^ii^—O9—H3	102.8
O8^i^—Ba1—O1^iii^	138.61 (12)	H2—O9—H3	100.9
O7—Ba1—O1^iii^	111.17 (9)	Ba1—O10—H4	122.3
O9—Ba1—O1^iii^	61.02 (9)	Ba1—O10—H5	113.6
O7^ii^—Ba1—O1^iii^	59.07 (10)	H4—O10—H5	107.6
O9^iii^—Ba1—O1^iii^	95.40 (9)	C5—N1—C1	124.8 (4)
O10—Ba1—O1^iii^	67.61 (11)	C5—N1—H1	117.6
O8^i^—Ba1—O5	64.24 (16)	C1—N1—H1	117.6
O7—Ba1—O5	93.23 (11)	O5—N2—O6	118.8 (4)
O9—Ba1—O5	111.61 (11)	O5—N2—C3	120.4 (4)
O7^ii^—Ba1—O5	94.27 (14)	O6—N2—C3	120.8 (4)
O9^iii^—Ba1—O5	77.39 (14)	O1—C1—N1	121.4 (4)
O10—Ba1—O5	137.72 (16)	O1—C1—C2	119.6 (4)
O1^iii^—Ba1—O5	152.77 (13)	N1—C1—C2	119.0 (3)
O8^i^—Ba1—O2	89.10 (10)	O2—C2—C3	128.6 (4)
O7—Ba1—O2	143.31 (9)	O2—C2—C1	114.2 (4)
O9—Ba1—O2	63.24 (9)	C3—C2—C1	117.1 (3)
O7^ii^—Ba1—O2	64.40 (9)	C2—C3—C4	122.6 (4)
O9^iii^—Ba1—O2	104.67 (9)	C2—C3—N2	118.4 (4)
O10—Ba1—O2	129.95 (11)	C4—C3—N2	119.0 (3)
O1^iii^—Ba1—O2	105.01 (9)	O3—C4—C3	127.7 (4)
O5—Ba1—O2	53.35 (10)	O3—C4—C5	114.3 (4)
O8^i^—Ba1—O2^iii^	121.74 (10)	C3—C4—C5	117.9 (3)
O7—Ba1—O2^iii^	64.65 (9)	O4—C5—N1	121.2 (4)
O9—Ba1—O2^iii^	111.41 (8)	O4—C5—C4	120.7 (4)
O7^ii^—Ba1—O2^iii^	84.77 (9)	N1—C5—C4	118.0 (3)
O9^iii^—Ba1—O2^iii^	61.45 (9)	O8—C6—O7	125.3 (4)
O10—Ba1—O2^iii^	67.23 (11)	O8—C6—C6^i^	117.0 (5)
O1^iii^—Ba1—O2^iii^	53.60 (8)	O7—C6—C6^i^	117.7 (5)
O5—Ba1—O2^iii^	135.85 (12)		

Symmetry codes: (i) −*x*, −*y*+1, −*z*+1; (ii) −*x*, *y*−1/2, −*z*+3/2; (iii) −*x*, *y*+1/2, −*z*+3/2.

### Hydrogen-bond geometry (Å, º)

**Table:**

*D*—H···*A*	*D*—H	H···*A*	*D*···*A*	*D*—H···*A*
N1—H1···O6^iv^	0.86	2.21	3.052 (5)	166
O9—H2···O3^v^	0.82	2.10	2.877 (4)	158
O9—H3···O8^iv^	0.82	1.84	2.639 (4)	163
O10—H4···O3^v^	0.82	2.14	2.828 (5)	141
O10—H4···O6^v^	0.82	2.30	3.010 (5)	146
O10—H5···O4^vi^	0.82	2.41	3.210 (5)	165

Symmetry codes: (iv) *x*, *y*−1, *z*; (v) *x*−1, *y*, *z*; (vi) *x*−1, *y*+1, *z*.
